# Multidrug-resistant organisms in patients from Ukraine in the Netherlands, March to August 2022

**DOI:** 10.2807/1560-7917.ES.2022.27.50.2200896

**Published:** 2022-12-15

**Authors:** Romy D Zwittink, Cornelia CH Wielders, Daan W Notermans, Nelianne J Verkaik, Annelot F Schoffelen, Sandra Witteveen, Varisha A Ganesh, Angela de Haan, Jeroen Bos, Jacinta Bakker, Caroline Schneeberger-van der Linden, Ed J Kuijper, Sabine C de Greeff, Antoni PA Hendrickx, on behalf of the Dutch CPE and MRSA Surveillance Study Groups

**Affiliations:** 1 Centre for Infectious Disease Control (CIb), National Institute for Public Health and the Environment (RIVM), Bilthoven, the Netherlands; 2 SWAB Working Group Surveillance of Antibiotic Resistance, Department of Medical Microbiology and Infectious Diseases, Erasmus University Medical Center, Rotterdam, the Netherlands; 3 The members of the network are acknowledged at the end of the article

**Keywords:** antimicrobial resistance, patients from Ukraine, CPE, CPPA, MRSA, CRAB

## Abstract

Since March 2022, there has been an emergence of multidrug-resistant organisms (MDRO) in the Netherlands in patients originating from Ukraine (58 patients, 75 isolates). For about half of these patients, recent hospitalisation in Ukraine was reported. Genomic surveillance revealed that the majority of the MDRO represent globally spread epidemic lineages and that 60% contain New Delhi metallo-β-lactamase (NDM) genes. Professionals should be aware of an increase in such MDRO associated with migration and medical evacuation of people from Ukraine.

The Netherlands is a country with a low prevalence of multidrug-resistant organisms (MDRO), including carbapenemase-producing Enterobacterales (CPE), carbapenemase-producing *Pseudomonas aeruginosa* (CPPA), carbapenem-resistant *Acinetobacter baumannii* complex (CRAB) and meticillin-resistant *Staphylococcus aureus* (MRSA) [1-4]. Active MDRO screening and infection prevention measures (e.g. isolation of suspected colonised patients) applies to all patients admitted to a Dutch hospital who have recently been admitted to a hospital abroad (< 2 months ago for > 24 h) or resided in a centre for asylum seekers. Since March 2022, there has been an emergence of MDRO in the Netherlands in persons originating from Ukraine. Persons from whom isolates were obtained were receiving hospital care in the Netherlands and are henceforth referred to as patients. Here, we describe the epidemiology and genetic analysis of MDRO from patients from Ukraine in the Netherlands.

## Multidrug-resistant organisms in the Netherlands and Ukraine

In the Netherlands, the prevalence and spread of MDRO are monitored through national surveillance of CPE (since 2011), CPPA (since 2020), CRAB (since August 2022) and MRSA (since 1989), and through the mandatory notification of CPE (since July 2019). Starting from March 2022, the war in Ukraine led to migration of people from Ukraine and medical evacuation of more than 1,000 patients from Ukrainian hospitals to hospitals in other countries, including the Netherlands [5]. The European Centre for Disease Prevention and Control (ECDC) advised to pre-emptively isolate patients transferred from hospitals in Ukraine or with a history of hospital admission in Ukraine in the last 12 months and to screen for carriage of MDRO [6]. Available information from Ukrainian military and general hospitals indicates high prevalence of MDRO in Ukraine between 2014 and 2021 [3,7,8]. Percentages of resistance of 17–84% against third-generation cephalosporins and carbapenems among Enterobacterales and *P. aeruginosa*, and > 50% resistance to carbapenems, fluoroquinolones and aminoglycosides among *Acinetobacter* species have been reported [3,7,8]. European surveillance data for Ukraine indicated 18% of *S. aureus* to be meticillin-resistant, while 41% meticillin-resistance was reported in healthcare-associated *S. aureus* infections [3,8]. However, these percentages are based on isolates from hospitalised patients and may therefore not be representative for MDRO carriage in the community [3,7].

Up to March 2022, the Dutch national surveillance did not detect any MDRO in patients from Ukraine. From 1 March to 31 August 2022, 47 CPE, 12 CPPA, three CRAB and 13 MRSA isolates from 56 patients originating from Ukraine were submitted to the national surveillance (Figure, panels A-D), representing 21% (CPE), 52% (CPPA) and 1.2% (MRSA) of the total number of submitted isolates for each of these species during this period. In the mandatory CPE notification system, 39 patients from Ukraine were reported (Figure, panel E); for 37 of them, isolates had been submitted to the Dutch national surveillance for characterisation. Adding the two extra patients without submitted isolate, the total number of patients was 58. Isolates were characterised phenotypically (matrix-assisted laser desorption/ionisation time-of-flight mass spectrometry (MALDI-ToF), carbapenem inactivation method and Etest for meropenem) [2,9] and genetically (whole genome (wg) sequencing, multilocus sequence typing (MLST) and wgMLST) [10], and metadata were collected during submission for CPPA, CRAB and MRSA, or through the national mandatory notification system for CPE [1,4]. Isolates and notifications were considered Ukraine-associated when the epidemiological questionnaire indicated that the patient had recently been hospitalised in Ukraine (< 2 months ago for > 24 h) or had contact with a Ukrainian hospital or visited Ukraine < 1 year before sampling.

**Figure:**
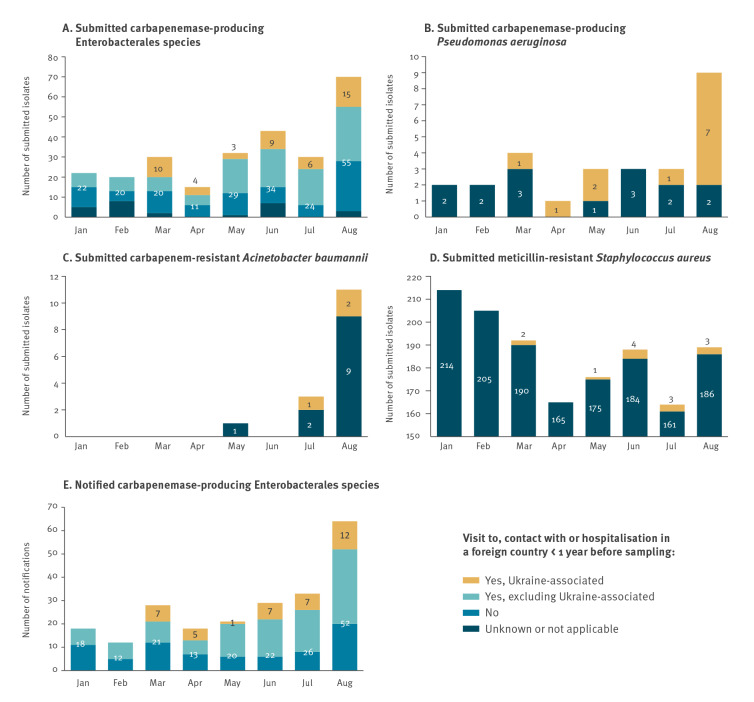
Number of submitted CPE (n = 262), CPPA (n = 27), CRAB (n = 15) and MRSA (n = 1,493) isolates, and mandatory CPE notifications (n = 223), the Netherlands, 1 January–31 August 2022

Numbers in the stacked bars indicate the total number of submitted isolates (panels A to D) or mandatory notifications (panel E) in a given category. For CPE submissions and notifications (panels A and E), isolates/notifications not associated with Ukraine are further stratified based on availability of data about recent travel/hospitalisation abroad in the year before sampling. The national CRAB surveillance (panel C) started on 1 Aug 2022.

## Epidemiology of patients from Ukraine with multidrug-resistant organisms

In the Netherlands, a risk factor for acquisition of MDRO is recent hospitalisation abroad [1,4]. We included a figure on mandatory CPE notifications from 2019 to 2022 in Supplementary Figure S1. In general, an increase in CPE notifications is observed in the third and part of the fourth quarter of the year, both in travel and non-travel-related CPE notifications. Also in 2022, CPE notifications increased, especially in August: 58 notifications compared with an average of 23 in the previous months (Figure, panel E) and compared with 28, 12 and 28 notifications in August 2019, 2020 and 2021, respectively. The observed increase in August 2022 was in part related to a larger number of patients for whom travel and/or hospitalisation abroad was reported (n = 38), of whom nine were patients from Ukraine, but also attributable to a larger number of patients with no record of travel or hospitalisation abroad (n = 20). There were no indications of geographical clustering or a local outbreak. 

Characteristics of patients from Ukraine with MDRO are shown in Table 1. The median age of patients was 33 years, and most were male. Except for CPPA and CRAB, the majority of MDRO were found in samples obtained for screening of patients considered at risk for MDRO because of recent hospitalisation or residing in a centre for asylum seekers. Twenty-one of the 39 patients with CPE had recently been hospitalised abroad. For CPPA, complete metadata were available for seven of 12 patients and six of them had been hospitalised abroad. For MRSA, complete metadata were available for 12 of 13 patients from Ukraine and for two patients, recent hospitalisation abroad was reported.

**Table 1:** Epidemiological characteristics of patients with CPE, CPPA, CRAB, MRSA and multiple MDRO originating from Ukraine, the Netherlands, sampled 1 March–31 August 2022 (n = 58)

Characteristic	CPE^a^ (n = 39)	CPPA^b^ (n = 12)	CRAB^b^ (n = 3)	MRSA^b^ (n = 13)	Multiple MDRO^a,b,c^ (n = 11)
	n	n	n	n	n
Median age in years (range)	34 (0–86)	31 (2–66)	45 (36–66)	27 (0–70)	28 (16–66)
Male	26	11	2	12	9
Female	13	1	1	1	2
Type of material					
Swabs from nose/throat/rectum/perineum	26	6	0	11	5
Wound/pus	10	6	3	2	5
Urine	3	0	0	0	1
**Additional metadata available**	**39**	**7**	**0^d^**	**12**	**11**
Reason for culturing					
Diagnostic/clinical indication	5	4	0	3^e^	2
Screening for MDRO, contact tracing or belongs to risk group	32	3	0	10^e^	8
Other/unknown	2	NC	NC	NC	1
Sampling location					
Outpatient clinic	11	0	0	4	1
Inpatient ward	24	7	0	6	9
Intensive care unit	3	0	0	2	0
Unknown	1	0	0	0	1
Invasive medical procedure/diagnostics					
Yes	22	1	0	NC	11
No	1	3	0	NC	0
Unknown	16	3	0	NC	0
Risk factor					
Hospitalisation abroad > 24 h during the previous 2 months	21	6	0	2	10

CPE: carbapenemase-producing Enterobacterales; CPPA: carbapenemase-producing *Pseudomonas aeruginosa*; CRAB: carbapenem-resistant *Acinetobacter baumannii*; MDRO: multidrug-resistant organism; MRSA: meticillin-resistant *Staphylococcus aureus*; NC: not collected.

^a^ Based on the notifications in the national mandatory notification system.

^b^ Based on the metadata entered in the database of the Dutch national surveillance.

^c^ These patients are also taken into account in columns ‘CPE’, ‘CPPA’ and ‘MRSA’, where applicable.

^d^ For CRAB, additional epidemiological data were not available because this pilot surveillance started only in August 2022.

^e^ One questionnaire was incomplete and only this question was available and therefore the denominator is 13 for this item.

For 11 patients (median age: 28 years; nine male), multiple MDRO isolates were submitted (Table 1). Six of the 11 patients had two different species of MDRO. Two patients carried three different MDRO: one carried one each of CPE, CPPA and CRAB and another carried two CPE and a CPPA. The remaining three patients each carried four different strains: one patient had three CPE and one CPPA, one patient had two CPE, one CPPA and one CRAB, and the final patient carried two CPE, one CPPA and one MRSA.

## Genetic analysis of multidrug-resistant organisms from patients from Ukraine

All submitted CPE, CPPA and CRAB isolates (n = 62, from 44 patients from Ukraine) produced carbapenemase as assessed by the carbapenem inactivation method; the individual results are provided separately in Supplementary Table S1 [9]. The majority of isolates (39/62; 63%) were resistant to meropenem according to the European Committee on Antimicrobial Susceptibility Testing (EUCAST) (minimum inhibitory concentration (MIC) > 8 mg/L [11]) as determined by Etest (bioMérieux). All submitted *Escherichia coli* isolates (n = 6) were susceptible with increased exposure for meropenem (MIC ≥ 2 and < 8 mg/L).

From 57 of the 62 isolates, Illumina next-generation sequencing (NGS) data were available. The majority of isolates were *K. pneumoniae* (37/62; 60%) and included globally spread MLST types ST307 (12/37), ST147 (10/37) and ST395 (7/37) [12,13], followed by *P. aeruginosa* (12/62; 19%), including ST1047 (5/12) and ST773 (4/12) [14], and *E. coli* (6/62; 10%) of varying STs including ST405 (2/6; Table 2) [15]. Three *K. pneumoniae* isolates belonged to ST23 and all CRAB isolates were from not yet assigned sequence types. 

**Table 2:** Carbapenemase-encoding genes and sequence types of multidrug-resistant organisms from patients from Ukraine in national surveillance, the Netherlands, sampled 1 March–31 August 2022 (n = 62)

Species and carbapenemase allele	Total	MLST sequence type														
		14	23	39	46	147	231	307	395	405	654	773	1047	5859	New ST^a^	Unknown^b^
***Acinetobacter baumannii***	**3**														**3**	
*bla*_OXA-23_, *bla*_OXA-66_	2														2	
*bla*_OXA-72_	1														1	
***Enterobacter cloacae* complex**	**1**						**1**									
*bla*_NDM-1_	1						1									
***Escherichia coli***	**6**				**3**					**2**					1	
*bla*_NDM-5_	4				3										1	
*bla*_OXA-244_	1									1						
*bla*_OXA-48_	1									1						
***Klebsiella pneumoniae***	**37**	**1**	**3**	**1**		**10**		**12**	**7**					**2**		**1**
*bla*_KPC-2_	1			1												
*bla*_KPC-3_	3							3								
*bla*_NDM-1_	16		2			5		6	3							
*bla*_NDM-1_, *bla*_OXA-232_	1													1		
*bla*_NDM-1_, *bla*_OXA-48_	8		1			3		2	1					1		
*bla*_NDM-9_	1							1								
*bla*_OXA-244_	1								1							
*bla*_OXA-48_	3					1			2							
*bla*_VIM-1_	1	1														
Unknown allele	2					1										1
***Proteus mirabilis***	**2**															**2**
*bla*_NDM-1_	2															2
***Providencia stuartii***	**1**															**1**
Unknown allele	1															1
***Pseudomonas aeruginosa***	**12**										**1**	**4**	**5**			**2**
*bla*_IMP-1_, *bla*_OXA-488_	4												4			
*bla*_IMP-10_, *bla*_OXA-488_	1												1			
*bla*_NDM-1_	4											4				
*bla*_VIM-2_	1										1					
Unknown allele	2															2
**Total**	**62**	**1**	**3**	**1**	**3**	**10**	**1**	**12**	**7**	**2**	**1**	**4**	**5**	**2**	**4**	**6**

MLST: multilocus sequence typing; ST: sequence type.

^a^ Unassigned sequence type.

^b^ Unsequenced isolates.

The CPE, CPPA and CRAB isolates from patients from Ukraine contained *bla*_NDM_-like carbapenemase alleles (37/62; 60%), followed by *bla*_OXA-48_ (12/62; 19%), *bla*_IMP_-like (5/62; 8%) and *bla*_KPC_-like alleles (4/62; 6%; Table 2). In contrast, 31% (76/242) of the other Dutch surveillance isolates (derived from persons sampled between 1 January and 31 August 2022) carried *bla*_NDM_-like carbapenemase alleles, followed by *bla*_OXA-48_ (70/242; 29%) and *bla*_OXA-181_ (25/242; 10%) (for each isolate, the detailed results from molecular analysis are provided in Supplementary Table S1 and S2). 

Sixteen of the 57 sequenced isolates from patients from Ukraine harboured combinations of two different carbapenemase alleles, of which *bla*_NDM-1_ with *bla*_OXA-48_ dominated (8/57). Significantly fewer (9%; 23/242; *p* < 0.0002) of the other Dutch surveillance isolates contained combinations of two different carbapenemase alleles, of which *bla*_OXA-23_ with *bla*_OXA-66_ (6/242; 2.5%) and *bla*_NDM‑5_ with *bla*_OXA-48_ (4/242; 1.7%) dominated. In Supplementary Figure S2, we append a resistome analysis using ResFinder v4.0 which revealed that MDRO from patients from Ukraine carried genes implicated in resistance towards more classes of antibiotics and disinfectants (p < 0.0001) than MDRO from the Netherlands.

We performed wgMLST using established in-house wgMLST schemes for *K. pneumoniae*, *E. coli*, *P. aeruginosa* [10,16] and *A. baumannii*, which indicated four potential transmission events of *K. pneumoniae* between patients from Ukraine and the Netherlands. Comparative wgMLST analysis of Dutch surveillance isolates with publicly available sequencing data from bacterial isolates from war-injured patients in military hospitals during the Eastern Ukraine conflict between 2014 and 2020 [7] indicates comparable sequence types (ST23, ST395, ST773), but no genetic clustering.

All MRSA isolates (n = 13, from 13 patients) carried *mecA,* and a small fraction harboured Panton-Valentine leukocidin (2/13). The NGS data of 12 MRSA isolates were available. Nine isolates belonged to six different MLST types and three to not yet assigned MLST types. Three isolates belonged to MLST ST5, two to ST22 and one of the isolates classified as livestock-associated MRSA (ST398). For each isolate, the detailed results from molecular analysis are provided in Supplementary Table S3. Based on wgMLST, there was no indication of MRSA transmission between patients from Ukraine and the Netherlands. No data on other MDRO are available since these are not monitored in the national surveillance.

## Discussion

We observed a marked increase in Ukraine-associated CPE in August 2022, which added to the non-Ukraine-associated, travel and non-travel related increase in CPE at that time. Given the ongoing routine infection prevention measures, it is unlikely that this is a result of a change in screening practices. Potential transmission of *K. pneumoniae* between patients from Ukraine and the Netherlands occurred only sporadically and outbreaks were not observed, an indication that infection prevention measures performed in hospitals in the Netherlands are adequate. About half of the patients from Ukraine with MDRO and with available additional metadata had recently been hospitalised in Ukraine. Besides, in patients from Ukraine with CPE, CPPA and CRAB, combinations of two carbapenemase alleles occurred significantly more frequently than usual in the Netherlands [1,2]. This is probably attributable to the medical evacuation of patients from Ukrainian hospitals. OXA-type and NDM-1 have been reported as the most frequent alleles among carbapenemase-positive MDRO causing hospital-acquired infections in Ukraine, but carbapenemase allele combinations were not reported [8]. Limitations of our study are the lack of denominator data, including how the number of MDRO from patients from Ukraine relates to the total number of patients, the monthly number of hospitalisations in the Netherlands, and how many patients from Ukraine have been screened for MDRO or how many were not colonised with an MDRO. In addition, information on war-related injuries, other clinical data of the patients and the timing of medical evacuations is missing. 

## Conclusion

We observed an increase in MDRO from globally epidemic high-risk sequence types retrieved from patients from Ukraine in the Netherlands. About half of these patients had recently been hospitalised in Ukraine. Eleven patients carried multiple MDRO. The high percentage of isolates containing *bla*_NDM_-like genes in their genomes, and the observation that the isolates are potentially resistant towards multiple classes of antibiotics, imply that infections with these bacteria are difficult to treat. Additional phenotypic resistance patterns of these MDRO need to be determined, including novel combinations of antibiotics, to provide physicians with therapeutic options for treatment of infections with carbapenemase-producing Gram-negative bacteria in patients from Ukraine. Healthcare professionals should be aware of the possible presence of these microorganisms when treating hospitalised patients from Ukraine and take adequate infection prevention measures to prevent the spread of these MDRO. 

## Supplementary Data

149000 Supplement
